# A flexible mixed‐data model applied to claims data for post‐market surveillance of prescription drug safety behavior

**DOI:** 10.1002/pst.2213

**Published:** 2022-04-03

**Authors:** Harris Butler, John D. Rice, Nichole E. Carlson, Elaine H. Morrato

**Affiliations:** ^1^ Biostatistics and Informatics, Colorado School of Public Health University of Colorado Anschutz Medical Campus Aurora Colorado USA; ^2^ Parkinson School of Health Sciences and Public Health Loyola University Chicago Chicago Illinois USA; ^3^ Health Systems, Management, and Policy, Colorado School of Public Health University of Colorado Anschutz Medical Campus Aurora Colorado USA

**Keywords:** FDA, joint, key performance indicators, mixed data, mixture, modeling, prescribing, REMS, risk mitigation, safety surveillance

## Abstract

We develop a new modeling framework for jointly modeling first prescription times and the presence of risk‐mitigating behavior for prescription drugs using real‐world data. We are interested in active surveillance of clinical quality improvement programs, especially for drugs which enter the market under an FDA‐mandated Risk Evaluation and Mitigation Strategy (REMS). Our modeling framework attempts to jointly model two important aspects of prescribing, the time between a drug's initial marketing and a patient's first prescription of that drug, and the presence of risk‐mitigating behavior at the first prescription. First prescription times can be flexibly modeled as a mixture of component distributions to accommodate different subpopulations and allow the proportion of prescriptions that exhibit risk‐mitigating behavior to change for each component. Risk‐mitigating behavior is defined in the context of each drug. We develop a joint model using a mixture of positive unimodal distributions to model first prescription times, and a logistic regression model conditioned on component membership to model the presence of risk‐mitigating behavior. We apply our model to two recently approved extended release/long‐acting (ER/LA) opioids, which have an FDA‐approved blueprint for best prescribing practices to inform our definition of risk‐mitigating behavior. We also apply our methods to simulated data to evaluate their performance under various conditions such as clustering.

## 1 INTRODUCTION

Title IX of the Food and Drug Administration Amendments Act (FDAAA) established the authority of the FDA to require Risk Evaluation and Mitigation Strategies (REMS) on the prescription and use of high‐risk drugs. REMS for the highest risk drugs include Elements to Assure Safe Use (ETASU). Such drugs include opioids for pain management, buprenorphine for opioid dependence, isotretinoin for acne, clozapine for schizophrenia, and riociguat for pulmonary hypertension.^1^ Because a REMS drug has serious risks, it is important to conduct active independent post‐marketing surveillance to ensure public safety.^2^, ^3^ The FDA is encouraging the research community to develop novel methods for assessing REMS to improve post‐market assessment of effectiveness of risk mitigation strategies.^4^ Thus, it is of interest to quantify the prescribing and risk mitigation behavior of existing drugs with the goal of developing a statistical analysis framework for near real‐time surveillance monitoring of these and new drugs that enter the market.

Medical and pharmacy claims data, such as the all payers claims data (APCD), are an important population‐based data resource useful for quantifying prescribing behavior and REMS adherence to certain ETASUs in existing drugs and continued monitoring over time. They are designed to inform cost containment and quality improvement efforts. Payers include private health insurers, Medicaid, children's health insurance and state employee health benefit programs, prescription drug plans, dental insurers, self‐insured employer plans and Medicare (where it is available to a state).^5^ However, administrative claims data present several statistical challenges. The outcomes of interest are multivariate including, but not limited to, prescription fill dates, doses, and quantities, as well as the completion of blood or pregnancy tests prior to prescribing ETASUs. This type of data is often termed mixed data^6^ where some outcome measures are continuous, such as times, weights, or heights, and some outcomes are discrete, such as success/failure, counts, or disease stages. These outcomes may not be independent. For example, a very restrictive REMS may correlate with a delayed adoption by prescribers or a low prescribing rate.

Incorporating these relationships via a mixed data model may be useful but most analyses of prescribing to date consider univariate responses.^3^, ^7^, ^8^, ^9^, ^10^ In addition, prescribing rates often have complex patterns over time due to unknown marketing efforts, policy changes, different user or prescriber subpopulation.^11^ This can result in a mixture or multimodal prescribing distributions where the factors defining the mixing components are often unknown for analysis. Thus, a highly flexible model of prescribing times is desired. Some innovative models for prescribing already exist^7^, ^8^ but they do not accommodate *unknown* heterogeneous subpopulations, nor do they accommodate joint modeling of multiple outcomes. Rather than attempt to bend our application around an unsuitable prescribing model, we attempt to combine mixed data modeling methods to create a prescribing model suitable for our application.

If the response vector includes both continuous and discrete domains, then the data are said to be mixed. A commonly studied type of mixed data, and relevant to our application, has one continuous response and one binary response. De Leon and Chough^12^ cover many approaches and are a valuable reference. Cox and Wermuth^13^ suggested an approach with a marginal model for the continuous response and a conditional model for the categorical response. Fitzmaurice and Laird^14^ use a marginal model for the categorical response and a conditional model for the continuous response. Catalano and Ryan^15^ treat the discrete portion as a censored observation of a continuous latent variable and fit their data with a correlated probit model. De Leon and Wu^6^ also use a continuous latent variable for the discrete response but use a copula to model the covariance between the components of the response. All of these approaches have useful features but none accommodate a heterogeneous population.

This work has two methodologic contributions: (1) we develop a joint model of prescribing times and REMS adherence and (2) we use a mixture model inside of our mixed data model for flexibly modeling the continuous data distribution and linking the continuous and discrete data.

More specifically, our model is based on conditional mixed data frameworks similar to the approaches of Cox and Wermuth^13^ and Fitzmaurice and Laird,^14^ where we develop a marginal distribution for the prescription fill date and a conditional distribution for REMS adherence. We investigate multiple distributions for the prescription fill date and flexibly model the complex patterns over time through mixture distributions. We then use the estimated mixture probabilities in our conditional model for REMS adheres, where the probability of being in a particular mixture component influences a patient's probability of being REMS adheres. We apply this framework to extended release/long‐acting (ER/LA) opioids that were approved after the FDAAA.

## 2 MOTIVATING EXAMPLE

In this paper, we develop a multivariate framework for modeling prescription behavior. We consider a multivariate response consisting of the first prescription time and whether a patient was REMS adherent. Because our response has a continuous component and a discrete component, our model must account for so‐called mixed data.^6^ Our model is described by a marginal distribution for the prescription fill date and a conditional distribution for REMS adherence. We then use the estimated mixture probabilities in our conditional model for REMS adherence, where the probability of being in a particular mixture component influences a patient's probability of being REMS adherent.

We chose to apply this framework to ER/LA opioids that were approved after the FDAAA and did not have any generics on the market.^16^ There have been several ER/LA opioids approved since the FDAAA. We focus our analysis on two of these drugs. The first is Belbuca (buprenorphine buccal film), which we chose due to its unique indication for pain management and opioid addiction.^17^ The second is Xtampza ER (oxycodone extended‐release capsules), which we chose due to its large prescription count.^18^ They can be considered in isolation because the lack of generics means there are no equivalent drugs to consider, and they were approved with a REMS already in place, so there is no difference between the drug approval time and the time the REMS started applying to them.

### 2.1 Prescription modeling and REMS adherence

Prescription drug behavior has been studied from several perspectives. Kalyanaram^9^ fits a linear regression model with log‐transformed responses and predictors to estimate the effect of direct‐to‐consumer advertising on prescription drugs. Cochran et al.^19^ use logistic regression to model the probability of opioid misuse. Zhang et al.^10^ consider whether characteristics of a first opioid prescription are associated with future high‐risk opioid use. Rigg and DeCamp,^20^ and Bavarian et al.^21^ use structural equation modeling to perform a causal analysis on factors related to opioid and stimulant misuse.

Some research has been conducted on how REMS policies affect drug prescribing. Blanchette et al.^3^ study REMS policy adherence for bosentan, a drug indicated for pulmonary arterial hypertension. They find that REMS adherence decreases as a patient receives increasingly more refills, and that patients were more likely to be REMS adherent immediately following the creation of the REMS. Their study takes time into account and considers important aspects of prescribing but relies on binning REMS adherence proportions and dates.

One recent area of prescription modeling applies the Bass model to prescription counts/times. Bass' original model^22^ was based on Rogers' Diffusion of Innovation theory.^23^ Vakratsas and Kolsarici^7^ use an extension of the Bass model to look at pent‐up and incident demand for newly marketed drugs. Dunn et al.^24^ fit the Bass model to a wide variety of drugs using prescription information from Australia's nationalized healthcare system.

Stovring et al.^25^, ^26^ consider how to estimate the distribution of prescription duration of use, even when information on the days supplied is not provided in the claim, as well as how to increase the accuracy of an estimated duration when days supplied is provided, but not assumed to be accurate. However, they only consider prescription duration and do not model any other aspect of prescribing behavior.

There are two main gaps in the existing literature for statistical modeling of prescribing behavior. First, most approaches conduct simple analyses, assuming that a linear model can adequately describe a heterogeneous population. Even when more sophisticated analysis methods are used, existing literature considers a univariate response. For example, Zhang et al.^10^ create a single binary response that aggregates several characteristics of high‐risk opioid use, when they could have conducted a multivariate analysis. Aggregating multiple criteria into a single response sacrifices the information on which aspect is responsible for making a prescription high‐risk. A joint model can consider multiple responses without having to aggregate and can account for correlation between the multiple responses.

## 3 MOTIVATING DATA

Our motivating data set is derived from the Colorado All Payer Claims Database (CO APCD), administered by the Center for Improving Value in Healthcare (CIVHC). The CO APCD includes administrative claims data for Coloradans covered by an insurance company that reports their information to CIVHC.^27^ Our data includes all claims from any patient who was prescribed any REMS drug between January 1, 2012 and July 31, 2019. The CO APCD has 68% coverage of all Coloradans, and 75% coverage of insured Coloradans.^27^ A summary of our data for Belbuca and Xtampza ER can be found in Table 1.

**TABLE 1:** Summary data for first prescriptions of buprenorphine buccal film and oxycodone ER

Selected ER/LA opioids		
	Buprenorphine buccal film	Oxycodone ER
*n*	305	1202
Marketing date	6/1/2015	5/10/2016
Appropriate first dose	45.9%	86.%
Opioid naive	0.0%	6.0%
Opioid tolerant	100%	44.7%
Medicare	27.5%	12.0%
Medicaid	26.9%	36.0%
Commercial	45.6%	52.0%
Avg. age (SD)	48.9 (13.4)	48.2 (14.1)
Female	70.1%	71.3%

*Note*: (CO APCD, 2012–2019).

For this cohort we consider a joint response made up of the first prescription time of a drug for each patient and the presence of risk‐mitigating behavior (i.e., an appropriate dosage as directed by the drug label). We define first prescription time as the number of days between the drug's initial marketing and when a patient filled their first prescription of that drug. The inclusion criteria for these cohorts encompass any Colorado patient with 6 months of insurance eligibility from any payer in the CO APCD prior to their first prescription claim of an extended‐release opioid. We use a six‐month look back period with the methodology described by Zhang^10^ to be reasonably sure that the first prescription for a drug seen in our data is the patient's first prescription of that drug. Each opioid forms a separate cohort, but it is possible that a patient may appear in more than one cohort. We do not consider a pooled cohort because overlap between cohorts and the definition of a first prescription becomes more complicated when a patient has prescriptions for multiple drugs in the cohort.

To determine *appropriate use* we define the following for each patient. *Opioid tolerant* patients had at least 60 daily milligrams morphine equivalent (also called MME) for at least 7 days leading up to their first prescription of the listed drug.^28^ Patients with an appropriate first dose were nontolerant patients with a daily dose in accordance with a drug's labeling, or tolerant patients with daily doses below the maximum recommendation. *Opioid naive* patients did not have any opioid prescriptions within 60 days before their first prescription of the listed drug.^17^, ^18^ Note that it is possible for a patient to be considered not naive, but still not meet the criteria for opioid tolerant. We also include information on age and gender in Table 1.

## 4 METHODS

Mixture models can be used to model a distribution consisting of heterogeneous subpopulations, without requiring explicit knowledge regarding which subpopulation an individual belongs. Everitt^29^ is a valuable reference for such models. Mixture models are common in statistics but seem not to have been applied in prescription modeling, although the concept of different subpopulations does appear in Vakratsas and Kolsarici's dual market model^7^ as well as Stovring et al.'s work.^25^, ^26^

We describe our basic model and then possible extensions, including clustering and inclusion of covariates. Our basic model is specified with a continuous marginal response (*Y*) is and binary conditional response (*X*). We specify Y as a mixture of two positive unimodal distributions. We let a patient's component membership c influence the estimated probability of adherence, α. For simplicity we start by only allowing two components in the mixture distribution, so $P\left(c=1\right)=\alpha$ and $\left(c=2\right)=1-\alpha .$ Let $\left(X_{i},Y_{i}\right)$ be a set of observations on individual i, where $c_{i}$ is an indicator for which of two components that i belongs to, $c_{i}\in \left\{1,2\right\},$ and $\theta_{c_{i}}$ being our parameters for the chosen distributions. The distributions for components 1 and 2 are $F_{1}\left(\theta_{1}\right)$ and $F_{2}\left(\theta_{2}\right),$ with densities $f_{1}$ and $f_{2},$ and we require them to be continuous unimodal distributions with positive support. Our basic model can be written as:

$$Y_{i}∼\alpha F_{1}\left(\theta_{1}\right)+\left(1-\alpha \right)F_{2}\left(\theta_{2}\right),Y_{i}>0$$

$$X_{i}|Y_{i}∼\text{Bernoulli}\left(\text{logi}t^{-1}\left(\gamma_{1}\pi \left(Y_{i}\right)+\gamma_{2}\left(1-\pi \left(Y_{i}\right)\right)\right)\right),\gamma_{1},\gamma_{2}\in \mathbb{R}$$

$$\alpha =P\left(c_{i}=1\right)\forall i$$

$$\pi \left(Y_{i}\right)=P\left(c_{i}=1|Y_{i}\right)=\frac{\alpha f_{1}\left(Y_{i}\right)}{\alpha f_{1}\left(Y_{i}\right)+\left(1-\alpha \right)f_{2}\left(Y_{i}\right)}.$$

The value $\pi \left(Y_{i}\right)$ weights the component densities at $Y_{i}$ by the marginal probability of being in each component, α. This represents the conditional probability of being in the first component given $Y_{i}$ and makes $X_{i}$ dependent on $Y_{i}.$ We investigate three distributions for the mixture component distributions—Bass, lognormal, and Weibull. The density for the Bass distribution is,

$$f\left(y|p,q\right)=\frac{{\left(p+q\right)}^{2}}{p}\frac{e^{-\left(p+q\right)y}}{{\left(1+\frac{q}{p}e^{-\left(p+q\right)y}\right)}^{2}},$$

with p>0 called the coefficient of innovation and q≥0 called the coefficient of imitation.^22^

The density of the lognormal distribution is,

$$f\left(y|\mu ,\sigma^{2}\right)=\frac{1}{y\sqrt{2\pi \sigma^{2}}}e^{-\frac{{\left(\log y-\mu \right)}^{2}}{2\sigma^{2}}},$$

with the log‐mean μ R and the log‐variance $\sigma^{2}$ > 0.

The density of the Weibull distribution is,

$$f\left(y|k,\lambda \right)=\frac{k}{\lambda }{\left(\frac{y}{\lambda }\right)}^{k-1}e^{-{\left(\frac{y}{\lambda }\right)}^{k}},$$

with shape *k* > 0 and scale λ > 0. We suggest these distributions for their popularity in certain fields. The lognormal and Weibull distributions are familiar to most statisticians and are common choices for continuous distributions with positive support. The Bass distribution is less familiar to most statisticians but has been used to model prescription times in other pharmaceutical research.^7^, ^8^

While the Bass distribution is uncommon in statistics, it has some useful characteristics. One such characteristic is that the parameters are explainable‐ the coefficient of innovation, *p*, is the rate at which people are prescribed a drug that is independent of the ongoing number of prescriptions. The coefficient of imitation, *q*, is the rate at which people are prescribed a drug that depends on the current number of prescriptions, which can reflect increasing uptake as public knowledge about a drug increases. Another nice feature of the Bass distribution is that maximum density is easily calculated by,

$$\underset{y}{\text{argmax}}\;f\left(y|q,p\right)=\frac{\log\left(q/p\right)}{q+p},$$

Having a closed form for the maximum density simplifies the fitting of models with maximum likelihood.^22^

### 4.1 Interpretation

Some parameters may be of clinical importance to investigators. The mixture parameter α represents the proportion of prescriptions that came from component 1. The $\gamma_{1}$ and $\gamma_{2}$ are weights affecting the probability of risk mitigating behavior depending on whether a patient is in the first or second component, respectively.

The parameters of component distributions $F_{1}$ and $F_{2}$ offer insight into the shape of each component mixture, and a distribution may be chosen that offers the best fit, or one that provides a desired interpretation. The Bass parameters p,q individually represent the rates of innovation and imitation with the maximum density discussed above, but does not offer an easily calculated mean, and the variance does not have a closed form.^22^ The lognormal and Weibull distributions have easily calculated means and variances.

### 4.2 Maximum likelihood estimation

Maximum likelihood is used to estimate the model, and the “BFGS” method provided by the R software works well.^30^ For parameters that are constrained, we recommend transforming constrained parameters into an unconstrained space before fitting, rather than using constrained optimization techniques. The p,q parameters for the Bass, $\sigma^{2}$ for the lognormal, and k,λ parameters for the Weibull distribution are constrained to positive values and a log transform maps them to the real line. The mixture parameter α is restricted to the unit interval and a logistic transform maps it to the real line. Once a model structure is chosen, fitting with maximum likelihood is a matter of coding the likelihood function (constructed from the densities listed above) in a suitable programming language, and using a optimization routine to find the parameters that maximize the likelihood for the given data. As hinted above, the R language works well for this since it provides an mle() function that accepts a negative log‐likelihood function and provides convenient methods to extract parameter estimates, calculate Wald confidence intervals, and common diagnostics such as Akaike's Information Criterion.^30^

Estimation may be conducted with simultaneous maximum likelihood where the full likelihood for $Y_{i}$ and $X_{i}$ is used but can also be fit sequentially when it is hard to find good starting values. In a sequential fitting method, one would fit the marginal model for $Y_{i}$, then fit the conditional model for $X_{\mathrm{ij}}|Y_{i}$ using the parameters found in the marginal model for $Y_{i}$, and finally, those parameters are used as starting points for estimating with the full likelihood.

If a method is used that returns an estimated Hessian matrix for the (negative) log‐likelihood, approximate standard errors and Wald confidence intervals can be constructed with little extra computational cost.

### 4.3 Extension to include covariates

It may be desirable to allow key parameters to depend on a set of covariates. Covariates may be included in a standard linear predictor for components *F*_1_ and *F*_2_, but this is dependent on the distribution chosen. Because covariates may be different for each individual, a single mixture parameter α is no longer appropriate, and we must allow the mixture parameter to change with an individual's covariates. A model that allows $\alpha_{i}$ to depend on covariates $Z_{i}\;$ can be written:

$$Y_{i}∼\alpha_{i}F_{1}\left(\theta_{1}\right)+\left(1-\alpha_{i}\right)F_{2}\left(\theta_{2}\right),Y_{i}>0,$$

$$\alpha_{i}=\text{logi}t^{-1}\left(Z_{i}\beta^{\prime }\right),$$

$$X_{i}|Y_{i}∼\text{Bernoulli}\left(\text{logi}t^{-1}\left(\gamma_{1}\pi \left(Y_{i}\right)+\gamma_{2}\left(1-\pi \left(Y_{i}\right)\right)\right)\right),$$

$$\pi \left(Y_{i}\right)=P\left(c_{i}=1|Y_{i}\right)=\frac{\alpha_{i}f_{1}\left(Y_{i}\right)}{\alpha_{i}f_{1}\left(Y_{i}\right)+\left(1-\alpha_{i}\right)f_{2}\left(Y_{i}\right)}.$$

It is difficult to conduct an analysis of deviance on these models, as one might do with a generalized linear model, as it is not obvious how to formulate the saturated model when the mixture components are two‐parameter distributions. However, we can conduct a likelihood ratio test of nested models to determine if covariates or groups of covariates significantly improve the model fit, or other model selection criteria such as Akaike's Information Criterion for non‐nested models.

### 4.4 Extension to more than two components and covariates

The model can also be extended to include m components, which may be helpful with some drugs. We extend the notation from two components: Let $Y_{i}$ be the response from individual *i*, and c is an index representing which of m components that i belongs to, $c_{i}=1,2,\ldots ,m.$ The marginal distribution is $Y_{i}∼\sum_{k=1}^{m}\alpha_{\mathrm{ki}}F_{k}\left(\theta_{\mathrm{ki}}\right)$, with $\alpha_{\mathrm{ki}}$ representing the probability of being in a particular component $\alpha_{\mathrm{ki}}=P\left(c_{i}=k\right),\sum_{j=1}^{m}\alpha_{\mathrm{ji}}=1\forall i$, and $\theta_{\mathrm{ki}}$ being a set of parameters for patient i that defines the distribution of the *k*th component distribution $F_{k}$. The conditional distribution of $Y_{i}$ given $c_{i}$ is then $Y_{i}|c_{i}∼F_{c}\left(\theta_{c_{i}}\;\right)$.

The single mixture parameter now becomes m−1 mixture parameters which must sum to 1. We formulate the mixture parameters similarly to multinomial logistic regression. The single γ parameter becomes a vector, allowing for each component to have a different probability of risk‐mitigating behavior.

$$Y_{i}∼\alpha_{1i}F_{1}+\alpha_{2i}F_{2}+\ldots +\alpha_{\mathrm{mi}}F_{m},\sum_{k=1}^{m}\alpha_{\mathrm{ki}}=1,\alpha_{\mathrm{ki}}>0\forall k,$$

$$\alpha_{\mathrm{ik}}=\frac{e^{Z_{i}\beta_{k}^{\prime }}}{1+\sum_{c=1}^{m-1}e^{Z_{i}\beta_{c}^{\prime }}},k=1,2,\ldots ,m-1,$$

$$\alpha_{\mathrm{ik}}=\frac{1}{1+\sum_{c=1}^{m-1}e^{Z_{i}\beta_{c}^{\prime }}},$$

$$X_{i}|Y_{i}∼\text{Bernoulli}\left(\text{logi}t^{-1}\sum_{k=1}^{m}\gamma_{k}\pi_{k}\left(Y_{i}\right)\right),$$

$$\pi_{k}\left(Y_{i}\right)=\frac{\alpha_{k}f_{k}\left(Y_{i}\right)}{\sum_{c=1}^{m}\alpha_{c}f_{c}\left(Y_{i}\right)}.$$

The choice of the number of components is left to the researcher. This may be informed by prior knowledge about a drug's prescribing behavior, by visual inspection of the data, or by fitting and comparing models with different numbers of components. There are advantages and disadvantages to each approach. Choosing components based on prior knowledge may be appropriate when the researcher is confident in their knowledge of the process they are modeling, but may not be the correct choice if prescribing behavior is influenced by unknown factors. Choosing components by visual inspection of the data is appealing when the data show clearly separated components like with the applications shown below but may not result in choosing the too few components when they are not well separated. Choosing components based on model fit is a defensible approach but is time consuming and may not be necessary if the researcher's prior knowledge of the process is sufficient or if it is easy to visually identify the number of components.

### 4.5 Adjusting for clustering

Prescribing behavior is influenced by the prescriber, but most prescription data are patient level. This creates clustering among patients when a single prescriber writes prescriptions for multiple patients. Clustering may also occur at the clinic level due to organizational policies.

Clustering is not explicitly modeled in our framework, but we can adjust our standard error estimates using Huber's robust standard error,^31^ specifically using the clustering result described by Freedman.^32^ This improves the accuracy of estimated standard errors and the coverage of confidence intervals with low computational cost. As we will discuss in the simulation results, clustering needs to be quite strong before adjustment is necessary; if clustering is not strong then adjustment may not be worth the loss in efficiency.

## 5 SIMULATION STUDY

We simulated data to examine model behavior for situations in which we did not have data available, as well as to confirm that our model performs adequately when fitting on the data that we did have. Our simulations matched our base model, as well as extensions to include a single covariate and clustering. Our generation process for the basic simulation was:

- For each patient i=1,2,…,100 and repeating with the Bass, lognormal, and Weibull distributions for $F_{1}$ and $F_{2}:$

1. Simulate component membership 1 versus 2 with *P*(*i* in component a) = α using a Bernoulli trial.
2. Simulate a first prescription time $Y_{i}$ from $F_{1}$ or $F_{2}$ conditioned on (a).
3. Simulate the presence of risk‐mitigating behavior according to,

$$P\left(X_{\mathrm{ij}}|Y_{i}\right)=\text{logi}t^{-1}\left(\gamma_{1}\pi \left(Y_{i}\right)+\gamma_{2}\left(1-\pi \left(Y_{i}\right)\right)\right).$$

After simulating the data, we fit three models to each data set using the Bass, lognormal, and Weibull distributions for $F_{1}$ or $F_{2}.$ Fitting three models to each data set enabled us to compare the sensitivity to using different distributions that are qualitatively similar‐ strictly positive and unimodal. As seen in the results later, the model in which the fitted distributions matched the generating distribution usually has the highest likelihood, but a researcher may choose to use a particular distribution to enable a particular interpretation. Since the distributions studied here were all two‐parameter distributions they could be directly compared with their model likelihood. When comparing models with different numbers of parameters it would be more appropriate to use a measure that penalizes for the number of parameters, such as Akaike's Information Criterion.

In a separate simulation we create data similar to the above simulation, but also created a single standard normal covariate for each patient and using that to inform the mixture parameter $\alpha_{i}$.

We also examined the effect that clustering had on standard error calibration by generating clustered data with a Gaussian copula.^33^ We simulated 300 patients in clusters of 10. Otherwise, our process for the cluster simulation was the same as our base simulation. Our correlation structure was compound symmetric within each cluster, with a correlation parameter of 0.9.

For the base, covariate, and clustered simulations, we conducted 1000 replications using a sample size of n=100 and the parameters listed in Table 2. The parameters for the distributions for Y were picked to deliver a mean of one for the first component and three for the second component, and standard deviation of 0.5 for both components. This is approximately the same scale as the prescription times (in years) observed in our motivating data. The parameters for α and γ were also selected to be approximately the same scale as observed in real data.

**TABLE 2:** Parameter values for simulations

Bass	Lognormal	Weibull
$p_{1}=0.0583$	$\mu_{1}=-0.112$	$k_{1}=2.38$
$q_{1}=4.36$	$\sigma_{1}^{2}=0.223$	$\lambda_{1}=1.15$
$p_{2}=3.86\times 10^{-5}$	$\mu_{2}=1.09$	$k_{2}=6.80$
$q_{2}=3.84$	$\sigma_{2}^{2}=0.0274$	$\lambda_{2}=3.24$
α=−0.5 (without covariate)		
$\alpha_{0}=-0.5$ (with covariate)		
$\alpha_{1}=1$ (with covariate)		
$\gamma_{1}=-2.5$		
$\gamma_{2}=-1\;\text{and}+1$		

## 6 RESULTS

Our simulation results show that this model performs well, with satisfactory bias and adequate coverage with the constructed confidence intervals. While fitting maximum likelihood models is not guaranteed to deliver unbiased estimates, parameter bias was always much smaller than the standard error even with a sample size of 100. For larger samples, maximum likelihood estimation will deliver smaller bias, making it of littler concern for real world applications. The highest likelihood model is usually found when the generating distributions for $F_{1}$ and $F_{2}$ match the distributions used for estimation. Table 3 shows the number of times that the estimating distribution family was the best fitting stratified by the generating distribution family.

**TABLE 3:** Model distributions with highest likelihood compared to the simulated distributions

	Highest likelihood distribution		
Simulated distribution	Bass	Lognormal	Weibull
Bass	1245	399	356
Lognormal	156	1548	296
Weibull	176	193	1631

However, the other distributions are often close in terms of fit, so an investigator may choose a slightly worse fitting model to gain the benefit of a desired parameter interpretation.

### 6.1 Standard error calibration

Our standard errors estimated using the Hessian matrix of the negative log‐likelihood are accurate for all parameters with independent samples. All coverages of 95% confidence intervals were near the desired 95% when clustering was not simulated. If strong clustering is present, we recommend adjusting the standard errors for clustering, discussed below. The coverage probabilities of 95% Wald confidence intervals are shown in Table 4.

**TABLE 4:** Coverage of 95% confidence intervals

	Generating distribution		
	Bass	Lognormal	Weibull
$p_{1}/\mu_{1}/k_{1}$	0.953	0.929	0.912
$q_{1}/\sigma_{1}^{2}/\lambda_{1}$	0.938	0.915	0.912
$p_{2}/\mu_{2}/k_{2}$	0.945	0.937	0.921
$q_{2}/\sigma_{2}^{2}/\lambda_{2}$	0.943	0.933	0.928
α	0.951	0.950	0.944
$\gamma_{1}$	0.966	0.966	0.963
$\gamma_{2}$	0.974	0.976	0.980

### 6.2 Adding a covariate

Introducing a covariate into the model did not change model fitting behavior. Even with a sample size of 100, the models fit well, giving unbiased estimates and accurately estimated standard errors.

### 6.3 Adjusting for clustering

Introducing strong clustering into the data did not hurt model fits very much when considering the point estimates, with no observable bias to the estimates. However, clustering causes biased naive estimated standard errors, which reduces coverage. Adjusting the standard error estimates with Huber's robust method results in improved coverage over the parameters influenced by clustering $\left(\alpha ,\gamma_{1},\gamma_{2}\right),$ but slightly worse coverage over the parameters not influenced by clustering. Results of coverage with and without adjustment for clustering are shown in Table 5. This behavior‐ an improvement in coverage for parameters affected by clustering and slight decrease in coverage for parameters without clustering‐ is well known for Huber's robust method.^32^

**TABLE 5:** Coverage of 95% confidence intervals using naïve/robust standard errors

	Generating distribution		
	Bass	Lognormal	Weibull
$p_{1}/\mu_{1}/k_{1}$	0.949/0.918	0.936/0.914	0.944/0.913
$q_{1}/\sigma_{1}^{2}/\lambda_{1}$	0.945/0.920	0.917/0874	0.932/0.926
$p_{2}/\mu_{2}/k_{2}$	0.951/0.931	0.942/0.934	0.943/0.927
$q_{2}/\sigma_{2}^{2}/\lambda_{2}$	0.947/0.932	0.938/0.923	0.939/0.925
α	0.548/0.949	0.581/0.949	0.612/0.950
$\gamma_{1}$	0.586/0.861	0.608/0.868	0.575/0.859
$\gamma_{2}$	0.709/0.794	0.725/0.784	0.745/0.806

## 7 APPLICATION WITH SELECTED ER/LA OPIOIDS

We focus our analysis on two drugs, summarized in the Motivating Example section. The first is buprenorphine buccal film, due to its unique indication for pain management and opioid addiction. The second is oxycodone ER, due to its large prescription count. Both of these drugs were approved after the ER/LA Opioid REMS was implemented in 2012. In our application, we use a joint response $\left(X,Y\right)$. $Y_{i}$ is a continuous measure of the first prescription time of a drug for patient i, and $X_{i}$ is a binary response measuring whether we observe risk‐mitigating behavior. For both drugs, we define risk‐mitigating behavior as an appropriate dosage determined by the drug labeling. $Y_{i}$ is measured in years since a drug's initial marketing. The time scale can be whatever is interesting to the researcher‐ it could be chosen as months or days and an identical fit would be delivered with only the relevant parameters being scaled, but there are inappropriate time choices (seconds) which might cause precision issues with the computer. Since our binary response is modeled as 0 or 1, we prefer our time scale to be small single digit numbers as well. We model prescriptions of buprenorphine buccal film as a two‐component mixture and oxycodone ER as a three‐component mixture, and consider all three recommended distributions (Bass, lognormal, and Weibull) for mixture components. For both drugs we used insurance type as a categorical covariate, $Z_{i},$ coded as (1,0,0) when Medicare paid for the majority of a claim, (0,1,0) when Medicaid paid for the majority of the claim, and (0,0,1) when a commercial payer paid for the majority of the claim.

## 8 RESULTS

**FIGURE 1:**
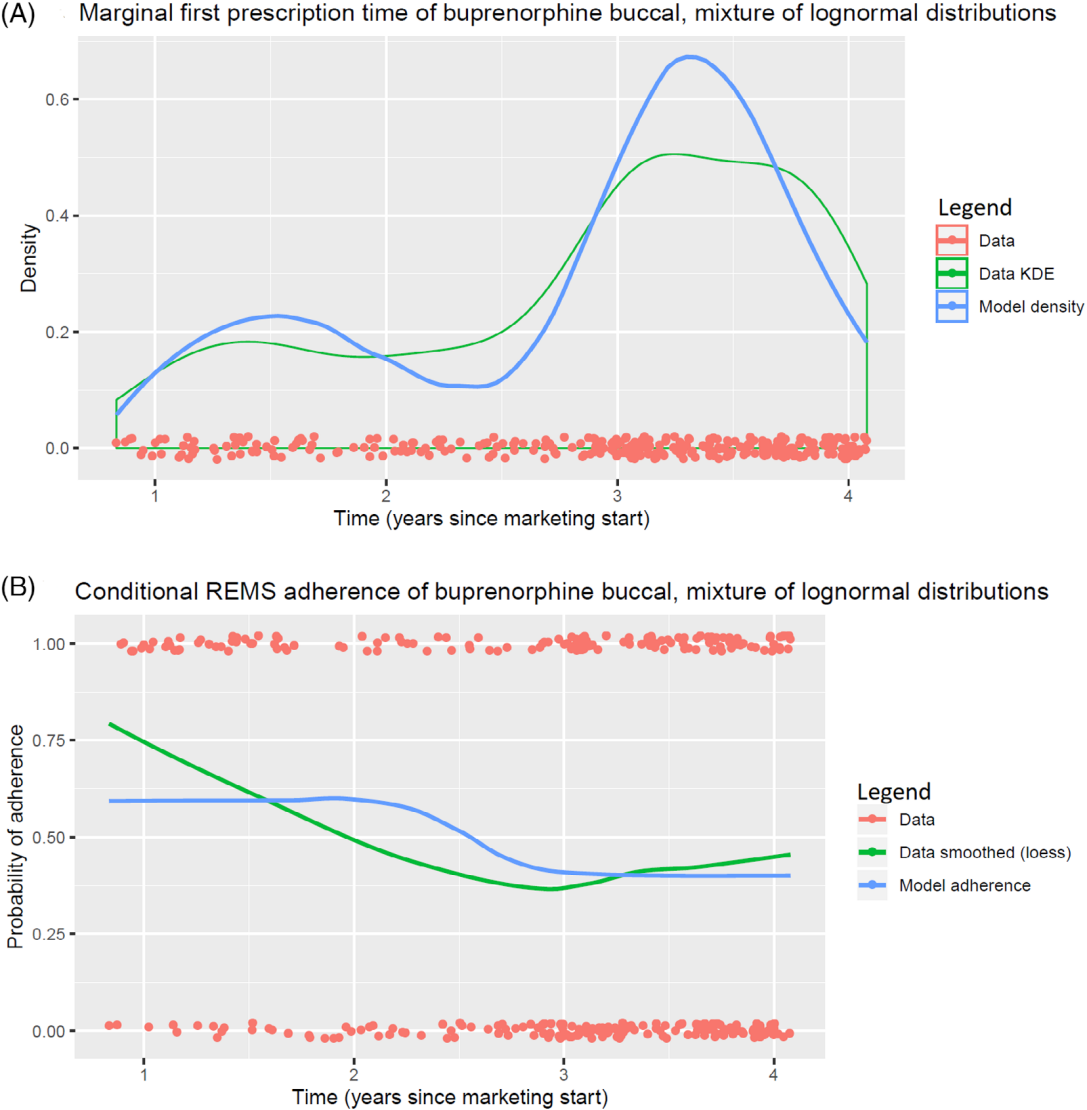
Model plots from fitting buprenorphine buccal film prescriptions with a mixture of lognormal distributions for first prescription time, and a conditional logistic regression for the probability of REMS adherence

**FIGURE 2:**
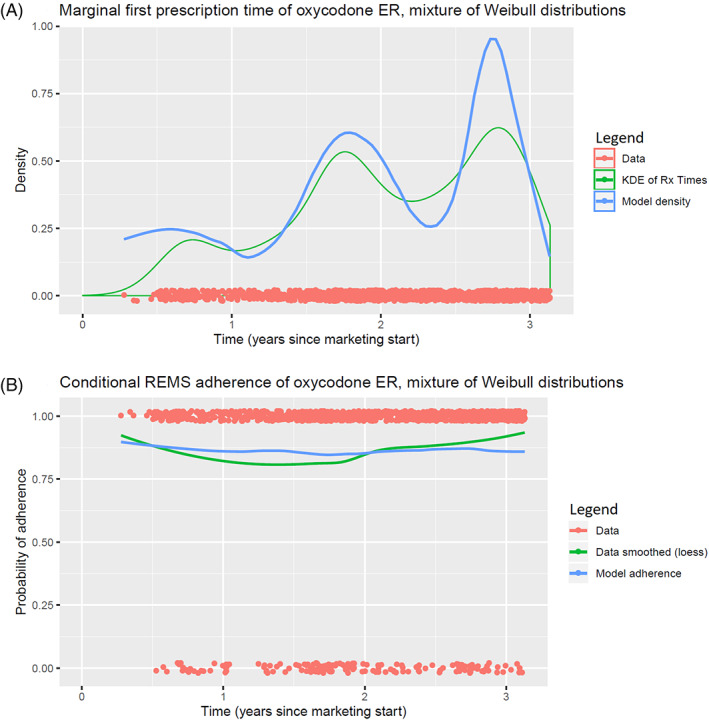
Model plots from fitting oxycodone ER prescriptions with a mixture of Weibull distributions for first prescription time, and a conditional logistic regression for the probability of REMS Adherence

We fit our model with maximum likelihood and our estimated parameters are shown in Tables 6 and 7. Plots of kernel density estimates of prescription times vs. our modeled densities and smoothed probabilities of risk‐mitigating behavior vs. our modeled probabilities are shown in Figures 1 and 2. Figure 1 comes from prescriptions of buprenorphine buccal film using a mixture of Weibull distributions for the first prescription times, with insurance type as a categorical covariate. Figure 2 comes from prescriptions of oxycodone ER using a mixture of Bass distributions for the first prescription times, with insurance type as a categorical covariate. We fit a two‐component mixture for buprenorphine buccal film and a three‐component mixture for oxycodone ER after visually inspecting our data.

**TABLE 6:** Estimated model coefficients for buprenorphine buccal film prescribing behavior

	Buprenorphine buccal film		
	Bass	Lognormal	Weibull
Log‐likelihood	−554.7	−552.2	−540.9
	$p_{1}=7.26\times 10^{-3}$	$\mu_{1}=0.531$	$k_{1}=4.29$
	$q_{1}=4.47$	$\sigma_{1}^{2}=0.127$	$\lambda_{1}=1.58$
	$p_{2}=6.95\times 10^{-5}$	$\mu_{2}=1.22$	$k_{2}=7.54$
	$q_{2}=3.24$	$\sigma_{2}^{2}=0.0152$	$\lambda_{2}=3.50$
$\beta_{1}=$	−0.829	−0.530	−0.847
$\beta_{2}=$	−1.11	−0.776	−1.12
$\beta_{3}=$	−1.98	−0.922	−2.03
$\gamma_{1}=$	−0.378	−0.420	−0.371
$\gamma_{2}=$	1.04	0.800	1.02

**TABLE 7:** Estimated model coefficients for oxycodone ER prescribing behavior

	Oxycodone ER		
	Bass	Lognormal	Weibull
Log‐likelihood	−1526.2	−1530.7	−1506.5
	$p_{1}=7.98\times 10^{-4}$	$\mu_{1}=-0.203$	$k_{1}=6.66$
	$q_{1}=14.0$	$\sigma_{1}^{2}=0.315$	$\lambda_{1}=0.725$
	$p_{2}=3.20\times 10^{-3}$	$\mu_{2}=1.87$	$k_{2}=5.09$
	$q_{2}=3.96$	$\sigma_{2}^{2}=0.162$	$\lambda_{2}=0.623$
	$p_{3}=4.96\times 10^{-10}$	$\mu_{3}=2.79$	$k_{3}=2.63$
	$q_{3}=8.45$	$\sigma_{3}^{2}=0.0688$	$\lambda_{3}=2.85$
$\beta_{11}=$	−0.0861	0.230	5.07
$\beta_{12}=$	−2.28	0.206	11.1
$\beta_{13}=$	−1.41	0.512	0.298
$\beta_{21}=$	0.566	0.459	2.27
$\beta_{22}=$	−0.406	−0.462	1.74
$\beta_{23}=$	0.911	0.868	0.562
$\gamma_{1}=$	5.70	5.08	5.71
$\gamma_{2}=$	0.867	0.558	0.805
$\gamma_{3}=$	2.39	2.30	2.30

Every model fit without difficulty using BFGS optimization, a quasi‐Newton method. All models fit similarly regardless of the component distribution used, although the Weibull distribution models had the highest likelihood for both drugs. We conducted likelihood ratio tests comparing intercept‐only models to the models that included insurance type (Medicare, Medicaid, or commercial). Insurance type was significant (*p* < 0.05) in every model, regardless of the component distribution.

In the models for buprenorphine buccal film prescription behavior, the coefficients were relatively consistent regardless of the component distributions used. That all β coefficients were negative indicate that all patients are more likely to be in the second component regardless of payer type, which we can see visually as the later mode in prescribing times being much larger than the first. Even though all patients are more likely to be in the later mode, the ranking of the β coefficients indicate that Medicare patients are more likely to be in the first component relative to Medicaid patients, and patients with commercial insurance are the least likely to be in the first component. That is, while a Medicare patient is overall more likely to be in the second (larger) component, the first (smaller) component is still made up of more Medicare patients because the $\beta_{1}$ is larger (less negative) than either $\beta_{2}$ or $\beta_{3}$. It is not clear why the insurer has a significant effect on when a patient might be prescribed a drug, but it is possible that the different payment options may have affected what type of therapy a prescriber considered for a patent. However, considering that we did not see the same pattern with oxycodone ER (below), this may not be the case. The γ coefficients are interpretable to tell us what the probability that a patient was prescribed an appropriate first dose conditioned on their component membership. A negative $\gamma_{1}$ indicates that patients in the first component have a more than 50% probability of being prescribed an appropriate first dose, and a positive $\gamma_{2}$ indicates that patients in the second component have less than 50% probability of being prescribed an appropriate first dose.

In the models for oxycodone ER prescription behavior, the β coefficients were not consistent across the component distributions used. While including insurance type was statistically significant, the inconsistent behavior depending on the choice of underlying distribution makes it difficult to offer a practical interpretation, as someone that prefers the Weibull distribution would have drawn different conclusions than someone that prefers the lognormal. Perhaps the best observation to make is that interpreting mixture component coefficients becomes more complex as the number of components increases, and it is important to keep the number of parameters small if interpreting parameters is important. The γ coefficients were consistent across models regardless of the β coefficients, and we can say that patients in the first component were the most likely to be prescribed an appropriate first dose, with patients in the second component being the least likely, but the positive values mean that patients had more than 50% probability of being prescribed an appropriate first dose regardless of which component they were in.

## 9 DISCUSSION

In this paper we introduce a modeling framework for multivariate modeling of prescribing behavior applicable for active population‐based surveillance of clinical quality improvement efforts such as FDA‐mandated REMS programs for promoting safe and appropriate use of prescription drugs.

Given prescribing patterns, we see that patient populations may be heterogeneous, which makes a mixture model appropriate for modeling first prescription times. Some aspects of prescription behavior, such as the presence of risk‐mitigating behavior, may depend on the component membership of each patient. Our model is flexible enough to accommodate covariates, arbitrary numbers of mixture components, and can adjust standard errors for clustering.

Our model fits our sample administrative claims data well and does not have problems with convergence or unreasonable estimates. Our simulations confirm that fitting the model with maximum likelihood delivers well‐behaved estimates. We also see that covariates can be included, and that adjusting standard errors for clustering using Huber's robust standard error method works reasonably well. While this framework may not be suitable for every drug, it seems to be appropriate for drugs that appear to be prescribed in waves. We do not know for sure why some drugs have this prescription pattern we suspect that these patterns are influenced by marketing and prescribers' awareness of the drugs as opposed to a corresponding pattern in new diagnoses for which the drugs are indicated.

For the drugs in our example, we see results that are similar to other research regarding REMS adherence. The characteristics of buprenorphine buccal film and oxycodone ER prescriptions make them much more likely to have high‐risk doses prescribed.^10^ Blanchette et al. showed that REMS policies can be around 70% even when there are safeguards in place, such as not filling the prescription until checking that a liver function test was performed.^3^ There may be valid reasons for prescribing a dosage in excess of the labeling, but our data showed that less than half of first buprenorphine buccal film prescriptions have an appropriate first dose according to the labeling‐ we do not want to speculate too much, but it is possible that there is either a common consideration not captured by the labeling or an actual issue with the prescribing behaviors. Oxycodone ER, a much more visible and more often prescribed drug, had a higher proportion of first doses in accordance with the labeling.

There are some limitations with our framework that may be overcome in future work. First, this framework only allows for a bivariate model of prescribing behavior, with first prescription times and a binary indicator of risk mitigating behavior. Risk‐mitigating behavior, however, may not be binary, and a more general model framework could account for additional responses related to prescription or risk‐mitigation behavior. The main issue in a conditional framework is the increasing complexity and dimensionality as additional responses are considered. Second, while most diagnostic methods for maximum likelihood models can be applied, it is not clear how some diagnostic methods would translate, such as an analysis of deviance. Third, while not discussed much in this paper, we would like to develop this framework to enable near real‐time analysis of administrative claims data and using a mixture model may not be feasible. By the time it becomes clear that a new mixture component should be added, the component likely should have been included much earlier; this may be problematic when attempting analysis in a timely fashion. Another limitation in this framework is that instability in parameter estimates such as the β coefficients may cause difficulty in interpretation, as one model suggest different results as another model that used a different distribution for the time to prescription. While this limitation is significant, there are some aspects that were consistent across models and can still provide value, such as the mean and variance of each component's prescription times and the probability of risk mitigating behavior in each component. Finally, covariates are only included in our framework by determining mixture component membership. We chose not to include covariates in the linear predictor for the probability of risk‐mitigating behavior because this would increase the dimensionality and likely have collinearity issues with $\pi \left(Y\right)$. A practitioner may choose to include covariates that affect the probability of risk‐mitigating behavior but should evaluate if the improvement in fit is worth the extra dimensionality and potential collinearity.

## Data Availability

The data that support the findings of this study are available from CIVHC, but the data use agreement between the authors and CIVHC restricts sharing, so are not publicly available.
